# Topical Photodynamic Therapy with Different Forms of 5-Aminolevulinic Acid in the Treatment of Actinic Keratosis

**DOI:** 10.3390/pharmaceutics14020346

**Published:** 2022-02-01

**Authors:** Joanna Bartosińska, Paulina Szczepanik-Kułak, Dorota Raczkiewicz, Marta Niewiedzioł, Agnieszka Gerkowicz, Dorota Kowalczuk, Mirosław Kwaśny, Dorota Krasowska

**Affiliations:** 1 Department of Cosmetology and Aesthetic Medicine, Medical University of Lublin, Chodźki 1 St., 20-093 Lublin, Poland; 2 Department of Dermatology, Venereology and Pediatric Dermatology, Medical University of Lublin, Staszica 11 St., 20-081 Lublin, Poland; vpaulinav@gmail.com (P.S.-K.); martan@akcja.pl (M.N.); agnieszka.gerkowicz@umlub.pl (A.G.); dorota.krasowska@umlub.pl (D.K.); 3 Department of Medical Statistics, School of Public Health, Center of Postgraduate Medical Education, Kleczewska 61/63 St., 01-826 Warsaw, Poland; dorota.bartosinska@gmail.com; 4 Department of Medicinal Chemistry, Faculty of Pharmacy, Medical University of Lublin, Jaczewskiego 4 St., 20-090 Lublin, Poland; dorota.kowalczuk@umlub.pl; 5 Institute of Optoelectronics, The Military University of Technology, Kaliskiego 2 St., 01-476 Warsaw, Poland; miroslaw.kwasny@wat.edu.pl

**Keywords:** photodynamic therapy, actinic keratosis, photosensitizer, 5-aminolevulinic acid

## Abstract

Photodynamic therapy (PDT) is safe and effective in the treatment of patients with actinic keratosis (AK). The aim of the study was to assess the efficacy, tolerability and cosmetic outcome of topical PDT in the treatment of AKs with three forms of photosensitizers: 5-Aminolevulinic acid hydrochloride (ALA-HCl), 5-Aminolevulinate methyl ester hydrochloride (MAL-HCl) and 5-Aminolevulinate phosphate (ALA-P). The formulations were applied onto selected scalp/face areas. Fluorescence was assessed with a FotoFinder Dermoscope 800 attachment. Skin areas were irradiated with Red Beam Pro+, Model APRO (MedLight GmbH, Herford, Germany). Applied treatments were assessed during the PDT as well as 7 days and 12 weeks after its completion. Ninety-four percent of patients rated obtained cosmetic effect excellent. The efficacy of applied PSs did not differ significantly. However, pain intensity during the PDT procedure was significantly lower in the area treated with ALA-P (5.8 on average) in comparison to the areas treated with ALA-HCl or MAL-HCl (7.0 on average on 0–10 scale). Obtained results show that ALA-P may undergo more selective accumulation than ALA-HCl and MAL-HCl. Our promising results suggest that PDT with the use of ALA-P in AK treatment may be an advantageous alternative to the already used ALA-HCl and MAL-HCl.

## 1. Introduction

Melanoma and non-melanoma skin cancer are the most common types of skin malignancies in the Caucasian population. Their incidence rates continue to rise, which is a matter of great concern to the patient and a substantial economic burden for the health care system [1,2]. Squamous cell carcinoma (SCC) deserves particular attention because of its tendency to metastasize. The risk of metastasis in invasive SCC is estimated to be about 4% and up to 2–3 times higher in immunosuppressed patients. Therefore, in addition to prevention, identification and therapy of early SCC is vital to avoid neoplastic progression [3].

One of premalignant conditions, with a malignant transformation rate ranging from 0.025% to 16%, is actinic keratosis (AK), an epidermal keratinocytic disorder induced by chronic exposure to ultraviolet (UV) light. A precise prediction of the risk of AK evolution into invasive SCC is infeasible [4]. However, it could be stated that AK plays an important role in the development of SCC. According to Criscione et al. [5], who studied a high-risk population to estimate the risk of progression of AK to SCC, approximately 65% of all primary squamous cell cancers developed from previously diagnosed AK. Moreover, subclinical and early AK lesions are also capable of direct transformation into invasive malignant disease [6]. All this information is sufficient to conclude that each detected AK lesion needs to undergo appropriate therapy, which is especially relevant to patients with multiple AK lesions in whom the risk of progression to SCC is higher [4,7].

Two separate treatment modalities are distinguished for AK, i.e., lesion-directed therapy and field-directed therapy [4]. The main focus of the latter includes treating subclinical lesions, reducing AK recurrence rates, and potentially lowering the risk of developing SCC. Application of the field-directed therapy is based on the theory of “field cancerization”, according to which the skin surrounding the lesions, due to chronic exposure to UV radiation, has the potential to transform to malignancy [8].

**Figure 1:**
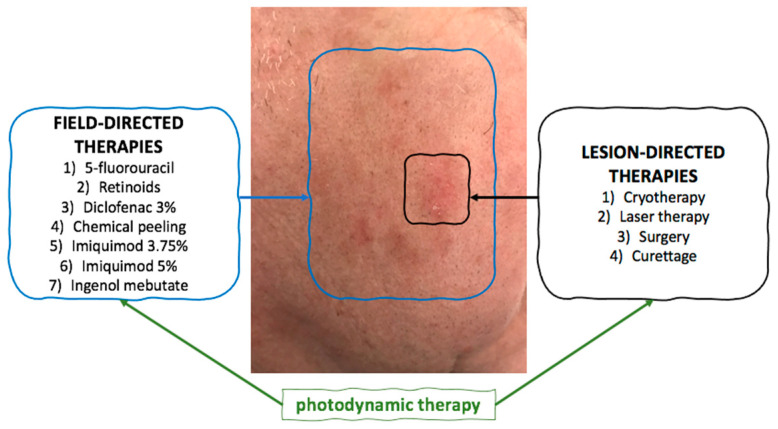
The methods of lesion- and field-directed therapies in actinic keratosis.

Photodynamic therapy (PDT) with the use of 5-aminolevulinic acid hydrochloride (ALA-HCl) as a photosensitizer (PS) and irradiation with blue light for the treatment of AK was approved by the FDA in the year 2000. In 2016, the FDA approved the use of ALA-HCl in combination with red light [8]. At present, due to the high treatment efficacy and a good cosmetic outcome, PDT is a routine, first-line treatment for AK. This type of therapy causes local and selective destruction of the neoplastic skin lesions without damage to the healthy tissue. PDT is a hardly invasive method, is generally well tolerated, is repeatable [9] and is applied either directly onto the lesion or onto the entire field of cancerization [4]. (Figure 1).

PDT has been shown to eliminate AK lesions and prevent their recurrence [8,10]. The action of PDT is based on selective photooxidation of the lesional tissue with the simultaneous involvement of three indispensable components, i.e., a PS, oxygen, and light of appropriate wavelength. The used PS accumulates in the dysplastic cells as well as in all the cells with high proliferation rates, where it is enzymatically metabolized via the heme pathway into the active endogenous photosensitizer protoporphyrin IX (PpIX). When the PS-treated skin is exposed to a light source that spans the absorption spectrum of PpIX (400–730 nm), the compound becomes photoactivated and triggers a photochemical reaction that generates cytotoxic singlet oxygen and free radicals, resulting in subsequent tissue loss [11,12]. The course of PDT and its efficacy are substantially dependent on PpIX production, distribution and depth of its penetration into the skin [13].

ALA-HCl and methyl aminolevulinate (MAL) are the most widely investigated PSs for the treatment of AK lesions. ALA-HCl and MAL are commercially available as Levulan^®^ and Metvix^®^, respectively [1]. There are reports of some disadvantages associated with these two PSs, especially pain experienced by patients during PDT, which seems to be the main adverse effect and a discouraging factor [14]. Therefore, new substances with more versatile properties are in the pipeline. Unlike ALA-HCl and MAL, the use of 5-aminolevulinic acid phosphate (ALA-P) has not yet been investigated as a PS in PDT of AKs.

The aim of the study was to assess the efficacy, tolerability and cosmetic outcome of topical PDT in the treatment of AK lesions with the use of three different forms of 5-ALA, i.e., 5-Aminolevulinic acid hydrochloride (ALA-HCl), 5-Aminolevulinate methyl ester hydrochloride (MAL-HCl) and 5-Aminolevulinic acid phosphate (ALA-P).

## 2. Materials and Methods

### 2.1. Study Group

Twenty-two Caucasian adults (of II and III skin phototypes), within the age range from 60 to 84 years, with multiple mild to severe AK lesions (Grade I-III according to Olsen) localized on the face and/or scalp were included in the study. The patients received PDT treatment at the outpatient clinic of the Department of Dermatology at the Medical University of Lublin, Poland between April 2018 and July 2021.

Inclusion criteria were as follows: multiple AK lesions Grade I-III according to Olsen and willingness to receive therapy and to participate in follow-up visits. Exclusion criteria were: pregnancy, epilepsy, history of photodermatosis, taking photosensitizing medication, and receiving any AK topical therapies at least three months prior to the beginning of the study. Written informed consent was obtained from all the patients before enrollment in the study. The study was approved by the local ethic committee (KE-0254/286/2019).

### 2.2. Study Formulations

In the study, 5-Aminolevulinic acid hydrochloride (ALA-HCl; 5-amino-4-oxopentanoic acid hydrochloride; C_5_H_10_ClNO_3_; [HOOC–CH_2_–CH_2_–CO–CH_2_–NH_3_^+^]Cl^−^); 5-Aminolevulinic acid phosphate (ALA-P; pentanoic acid, 5-amino-4-oxo-, phosphate (1:1); C_5_H_12_NO_7_P; [HOOC–CH_2_–CH_2_–CO–CH_2_–NH_3_^+^]H_2_PO_4_^−^); 5-Aminolevulinate methyl ester hydrochloride (MAL-HCl; methyl 5-amino-4-oxopentanoate hydrochloride; C_6_H_12_ClNO_3_; H_3_COOC–CH_2_–CH_2_–CO–CH_2_–NH_3_^+^]Cl^−^), obtained from Arisun chempharm Co., Ltd., Xi’an, China, were used PSs. The purity of ALA-HCl was 99.5% and of ALA-P was 99.2%, whereas MAL-HCl (purity of 97.0%) had to be further purified (recrystallization from methyl alcohol in the acidic environment) until its purity reached 99.5%. The purity of the used substances was confirmed with the standards by high-performance liquid chromatography (HPLC). The lipophilicity (LogP value) for the substances to be examined were calculated using the computer programs based on different calculation methods: ACD/LogP (Advanced Chemistry Development Inc., Toronto, ON, Canada; http://www.acdlabs.com (accessed on 17 January 2022)) ALogPs (VCCLAB, Virtual Computational Chemistry Laboratory, German Research Center for Environmental Health, Neuherberg, Munich, Germany; http://www.vcclab.org (accessed on 17 January 2022)) and miLogP (Molinspiration Software; http://www.molinspiration.com/cgi-bin/properties (accessed on 17 January 2022).

In order to obtain the study formulations, each of the three substances was added to the creamy LIPOBAZA. Since each formulation was to contain 10% of pure 5-ALA, the following concentrations of the study formulations were used: ALA-HCl—12.7%, MAL-HCl—12.5%, ALA-P—17.5%. The degree of homogenization was controlled with the use of an optical microscope.

### 2.3. Treatment Protocol

#### 2.3.1. Application of Study Formulations

**Figure 2:**
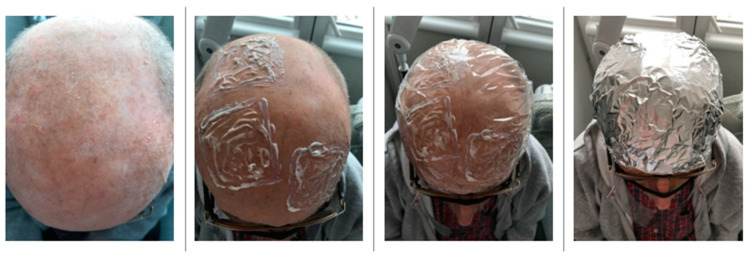
Application of study formulations in PDT of AK lesions.

In order to avoid the patients’ subjective pain assessment, after the scales and crusts were gently removed from a selected face or scalp field designated for the treatment, in each patient, the skin field to be treated was divided into three roughly equal areas onto which a 1 mm-thick layer of the ALA-HCl, MAL-HCl or ALA-P formulation was applied. Caution was taken to avoid overlapping and mixing of the used formulations. Finally, an occlusive plastic dressing and aluminum foil were placed over the treated skin surface. (Figure 2).

After a 3 h incubation period, the applied dressing and the remains of the study formulations were removed with 0.9% saline solution swabs.

#### 2.3.2. Assessment of Skin Fluorescence Following Application of Study Formulations

**Figure 3:**
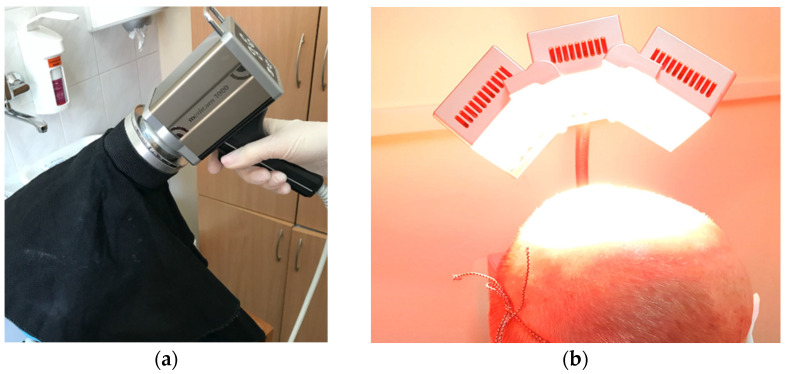
The use of FotoFinderDermoscope 800 to assess the fluorescence (**a**); the use of MedLight GmbH red light 630 ± 5 nm (**b**).

Qualitative absorption of the PSs contained in each of the study formulations was assessed with the use of a special attachment of FotoFinderDermoscope 800 with white and violet LED diodes emitting LED light. The opaque special material of the FotoFinder FD lens was used. (Figure 3a) The fluorescence coming from PpIX formed after application of each PS was rated as high, medium and low, as well as diffuse, confluent and blotchy.

#### 2.3.3. Irradiation with Red Light 630 ± 5 nm

The field-directed PDT was performed on the selected areas of the face and/or scalp. The study participants were instructed to wear protective glasses during irradiation.

Irradiation sessions were conducted with the use of Red Beam Pro+, Model APRO (MedLight GmbH, Herford, Germany), which insures optimal light penetration. (Figure 3b).

The Red Beam Pro+ lamp contains three movable units with 78 high power red light-emitting diodes, which provide red light operating at 630 ± 5 nm [9]. A total dose per session was 37 J/cm^2^, and the light power intensity was equal to 68 mW/cm^2^. The distance from the face and/or scalp was 10 cm. The patients were instructed to avoid sun exposure for 48 h following the treatment.

#### 2.3.4. PDT with Study Formulations Efficacy Assessment

Assessment of the percent of AK lesion clearance in each treated area was performed 12 weeks after PDT completion.

#### 2.3.5. PDT with Study Formulations Cosmetic Outcome and Patient Satisfaction Assessment

Twelve weeks after the PDT completion, cosmetic outcome assessment was performed with the use of a four-grade scale (Table 1), and the study participants were asked to express their level of satisfaction with the PDT and willingness to repeat the therapy if the need be.

#### 2.3.6. PDT with Study Formulations Tolerability Assessment

All the study participants were assessed by the same dermatologists during the procedures, shortly after their completion, and 7 days after the PDT completion.

During the procedure and 7 days after, the study participants were asked to evaluate pain intensity in each treated area on a 10-point VAS scale, and their exact location was determined with a pointer.

Frequency and severity of occurrence of PDT side effects, i.e., erythema, edema, desquamation and crusting, were defined as: none, mild, moderate, or severe.

Erythema and edema were assessed shortly after and 7 days after PDT.

Exfoliation, crusting and pigmentation were assessed 7 days after PDT.

Photographs of the skin lesions were taken at every visit.

### 2.4. Statistical Methods

The data were analyzed using STATISTICA 13 software (Statsoft, Kraków, Poland). The mean and standard deviation were estimated for numerical variables, as well as for absolute numbers (n) and percentages (%) of the occurrence of items for categorical variables.

Wilcoxon’s signed rank test for paired samples was used to compare severity of pain (10-point scale from 0 to 10) or clearance (in percentage) between every pair of two photosensitizers between the first and the second procedures.

The significance level was assumed to be 0.05 in all statistical tests.

## 3. Results

### 3.1. Characteristics of the Study Group

Characteristics of the AK patients and AK lesions are presented in Table 2.

Since extensive AK lesions are more frequently observed in males, we selected 21 men (18 with AK lesions on the scalp and 3 on the face) as well as 1 woman presenting with facial AK lesions.

Each of the study subjects completed the first PDT procedure, and because of extensive AK lesions, 16 of them qualified for a second PDT session. One patient refused to continue the treatment because of severe pain experienced during the first PDT procedure.

### 3.2. Fluorescence Assessment Following Application of Study Formulations

**Figure 4:**
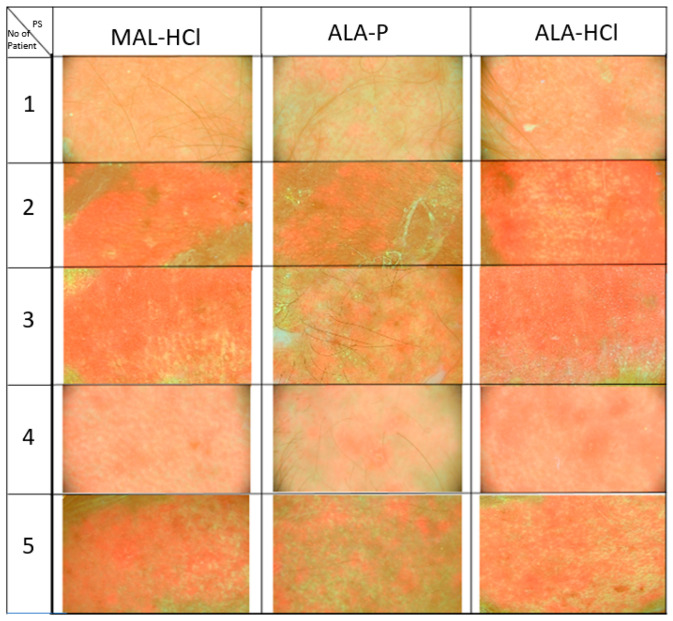
Intensity and distribution of fluorescence after application of ALA-HCl, MAL-HCl, and ALA-P.

Assessment of fluorescence intensity was performed for each of the study subjects before application of irradiation with red light 630 ± 5 nm. In the majority of patients (88% of them in the areas treated with ALA-HCl and 85% of them in the areas treated with MAL-HCl), the fluorescence intensity was high and confluent, while in the majority of patients (82% of them in the areas treated with ALA-P), the fluorescence intensity was lower and blotchy. (Figure 4).

### 3.3. PDT with Study Formulations Efficacy

**Figure 5:**
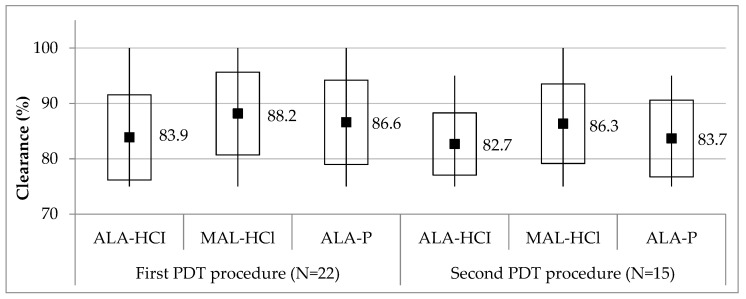
Clearance of actinic keratosis lesions 12 weeks after the first and second PDT procedures with the use of study formulations. Midpoint, mean; box, mean ± standard deviation; whiskers, min–max. Clearance did not significantly differ among three studied PSs as well as between the first and second procedures (*p* > 0.05). *p* stands for Wilcoxon’s signed rank test for paired samples.

Clearance 12 weeks after the first and second PDT procedures performed with ALA-HCl, MAL-HCl and ALA-P is presented in Figure 5. The clearance of AK lesions 12 weeks after the first PDT procedure in 22 patients was 83.9 ± 7.7%, 88.2 ± 7.5% and 86.6 ± 7.6% on average for ALA-HCl, MAL-HCl and ALA-P, respectively, while 12 weeks after the second PDT procedure in 15 patients, it was 82.7 ± 5.6%, 86.3 ± 7.2% and 83.7 ± 6.9%, respectively. The efficacy of any of the applied PSs did not differ significantly in the overall treatment of AK lesions (*p* > 0.05) both after the first and second PDT procedure.

### 3.4. PDT with Study Formulations COSMETIC Outcome and Patient Satisfaction

The vast majority of the study participants evaluated the overall cosmetic effect as excellent (94%), and the remaining 6% of them described it as good. Since scarring, atrophy, or induration were not observed, none of the participants found the PDT cosmetic outcome to be fair or poor. Patient satisfaction with the treatment was high, except for one patient who did not want to repeat the procedure because of excruciating pain experienced during the first PDT procedure.

### 3.5. PDT with Study Formulations Tolerability

During PDT, all the studied patients reported at least one adverse reaction. Pain was their most frequent complaint, and if it was unbearable, short breaks in irradiation were taken, or a cool dressing was applied.

**Figure 6:**
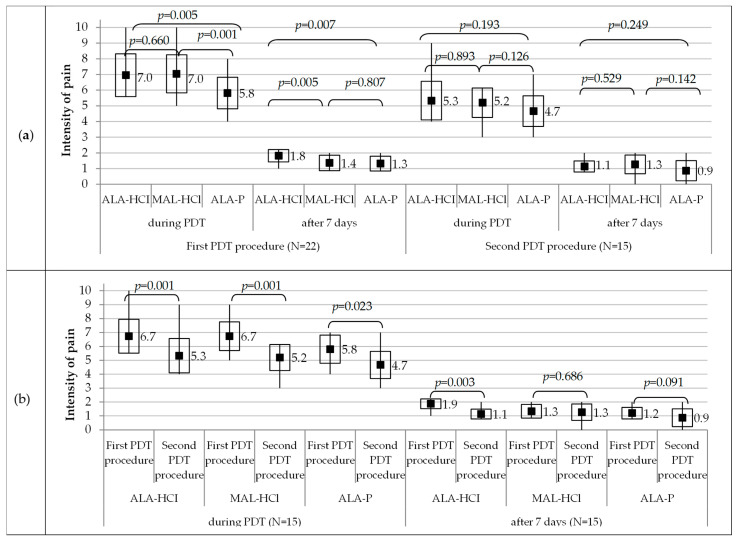
Pain intensity during PDT and 7 days after PDT in the first procedure (*n* = 22 patients) and in the second procedure (*n* = 15 patients): compared between every pair of two photosensitizers (**a**); compared between the first procedure and the second procedure (*n* = 15 patients) (**b**). Pain intensity on 10-point scale from 0 to 10. Midpoint, mean; box, mean ± standard deviation; whiskers, min–max. *p* stands for Wilcoxon’s signed rank test for paired samples.

Pain intensity (on 10-point scale from 0 to 10) during PDT and 7 days after the PDT completion was compared between the PSs grouped in pairs, in both the first and second procedures separately (Figure 6a).

In the 22 studied subjects, pain intensity during the first PDT procedure was significantly lower in the area treated with ALA-P (5.8 on average) in comparison to the areas treated with either ALA-HCl or MAL-HCl (7.0 on average in 0–10 scale). However, pain intensity 7 days after the first PDT procedure was significantly lower for MAL-HCl and ALA-P (1.4 and 1.3 on average, respectively) in comparison to ALA-HCl (1.8 on average).

In 15 patients, pain intensity during the second PDT procedure did not significantly differ between the three used PSs (5.3 on average for ALA-HCl; 5.2 on average for MAL-HCl; 4.7 on average for ALA-P). Pain intensity 7 days after the second PDT procedure was low and did not significantly differ between the three PSs (1.1 on average for ALA-HCl; 1.3 on average for MAL-HCl; 0.9 on average for ALA-P).

Pain intensity (on 10-point scale from 0 to 10) during PDT and 7 days after PDT was compared between the first and second procedures in 15 patients who underwent both procedures (Figure 6b).

Pain intensity was significantly lower during the second PDT procedure than during the first PDT procedure for each PS (*p* = 0.001 for ALA-HCl and MAL-HCl, *p* = 0.023 for ALA-P).

Pain intensity 7 days after the second procedure was significantly lower than 7 days after the first PDT procedure only in ALA-HCl (*p* = 0.003), while it was not significant in MAL-HCl (*p* = 0.686) or ALA-P (*p* = 0.091).

It was noted that the pain experienced during treatment tapered off/subsided together with the time of exposure to the red LED light.

**Figure 7:**
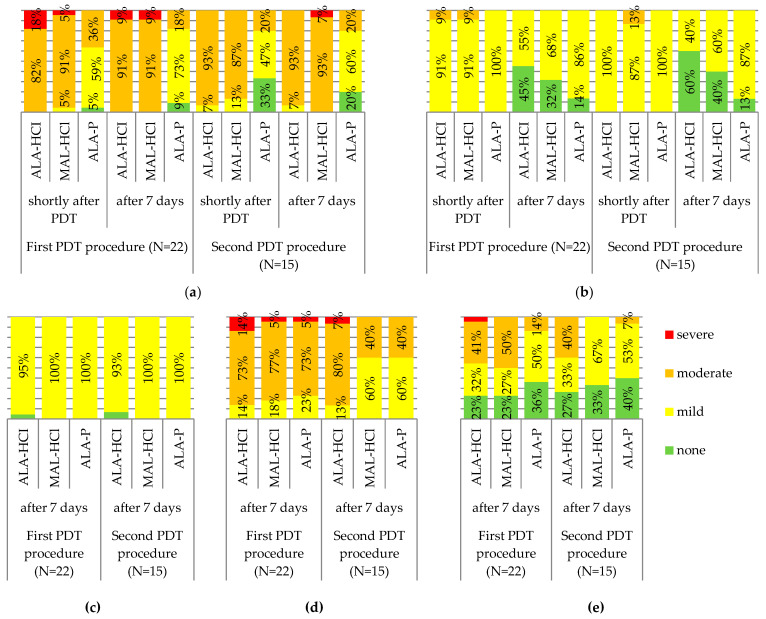
The severity of erythema (**a**), edema (**b**), desquamation (**c**), crusting (**d**), and pigmentation (**e**) in the first and second PDT procedures.

In our study, similar local responses to the three investigated PSs were observed. It was also observed that in the area treated with the ALA-P formulation, the intensity of erythema was mild in over half of the study participants (59%), while in the area where ALA-HCl and MAL-HCl were applied, the erythema intensity was moderate in the vast majority of the studied patients (82% and 91%, respectively). Seven days after the first PDT procedure, the same differences were still observed for the three studied PSs. No differences in erythema intensity were observed between the first and second PDT procedures, both shortly after and 7 days after PDT (Figure 7a).

Shortly after and 7 days after PDT completion, edema intensity was mild in all or almost all study subjects regardless of the applied PS during both first and second PDT procedures (Figure 7b).

Seven days after PDT completion, desquamation was mild in all the areas treated with MAL-HCl and ALA-P and in all but one patient treated with ALA-HCl, both in the first and second PDT procedures (Figure 7c).

Seven days after PDT completion, in three-quarters of the study participants, crusting intensity was moderate, regardless of the used formulation in the first PDT procedure, whereas in 80% of the patients, it was moderate in the area treated with ALA-HCl, and in 60% of the patients, it was mild in the areas treated with MAL-HCl and ALA-P (Figure 7d).

Seven days after PDT completion, the lowest pigmentation intensity was observed in the areas treated with ALA-P both in the first and second PDT procedures (Figure 7e).

**Figure 8:**
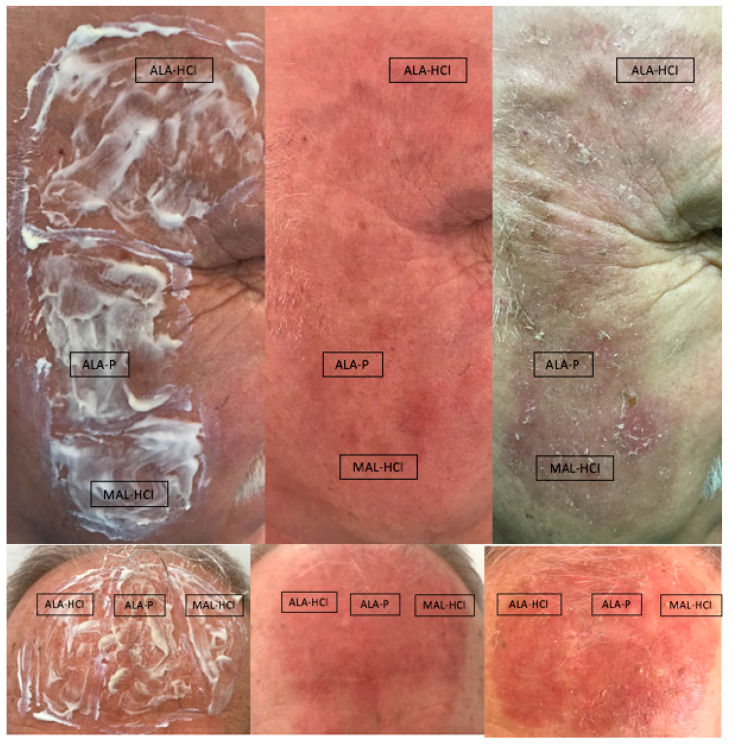
Local adverse reactions observed in the studied patients.

Some examples of local adverse reactions observed in the studied patients are presented in Figure 8.

**Figure 9:**
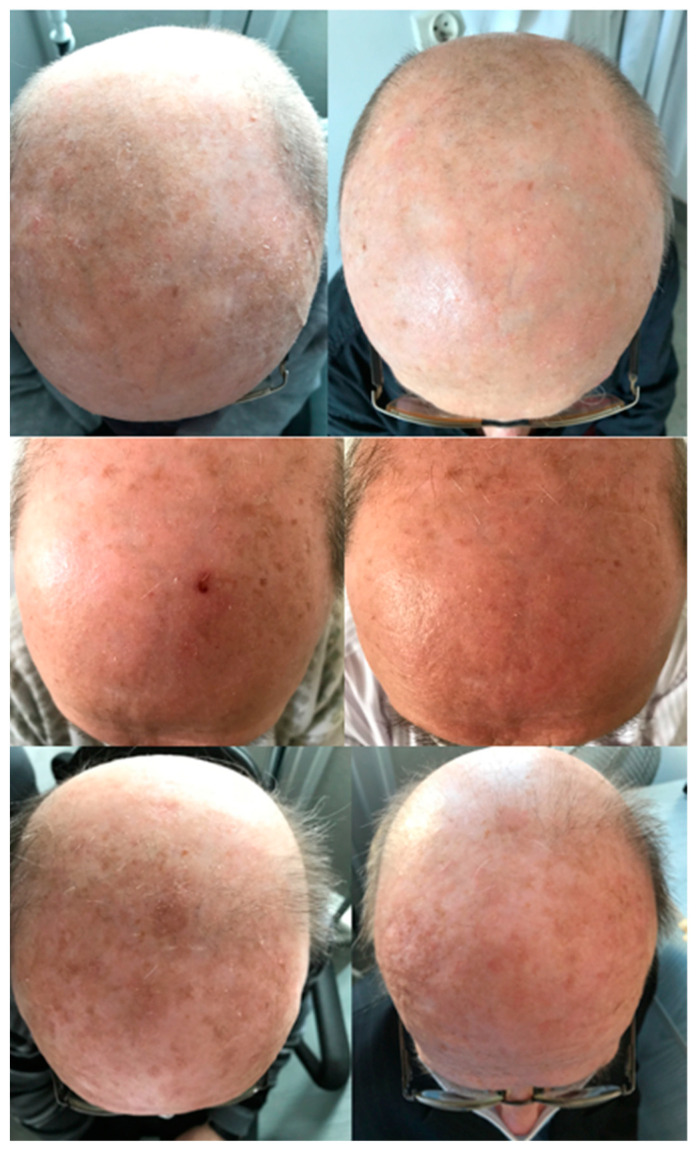
Comparisons of the skin areas in the studied patients before PDT and 12 weeks after procedure completion.

The comparisons of the skin areas in the studied patients before PDT and 12 weeks after procedure completion are shown in Figure 9 and Figure S1 (Supplementary Materials).

## 4. Discussion

In actinic keratoses, which are common skin lesions that may progress to invasive SCC, PDT is invaluable because of its minimal invasiveness and high efficacy. This paper presents the first observational, uncontrolled study of the efficacy and tolerability of a novel photosensitizer known as ALA-P and draws comparisons with two other commercially available PSs, i.e., ALA-HCl and MAL-HCl used in PDT of AK lesions. Aminolevulinic acid phosphate (C_5_H_12_NO_7_P), with a molecular weight of 229.13 g/mol, is a fairly new synthetic chemical compound also known as UNII-FM8DCR39GH; Pentanoic acid, 5-amino-4-oxo-, phosphate (1:1); 868074-65-1; 5-Aminolevulinic acid phosphate. Due to its properties, it has already been approved as a nutritional supplement in Japan and a few other Asian countries [15]. When administered by mouth, ALA-P has been shown to reduce blood glucose levels [16]. Furthermore, Higashikawa et al. observed that ALA-P diminished the severity of negative emotions in individuals continuously feeling physically fatigued [17]. All this made us presume that if orally used ALA-P is safe, effective and well tolerated, it could also be applied onto the skin with no need for in vivo tests to treat AK lesions with the use of PDT.

Thus, the novelty of the method presented in this study consists of the innovative, topical use of ALA-P as a photosensitizer, whereas assessment of the efficacy, tolerability and cosmetic outcome of ALA-P application in comparison with two other photosensitizers, i.e., ALA-HCl and MAL, revealed slightly better tolerance of the PDT procedure with the use of ALA-P.

The use of ALA-HCl and MAL in PDT has proven to be highly effective [18,19]. Nevertheless, the number of papers directly comparing the clinical outcomes of PDTs with ALA-HCl and MAL in AK patients is limited [18]. Moloney et al. performed a randomized, double-blind, prospective study comparing the efficacy and adverse effects of MAL-PDT and ALA-PDT in the treatment of scalp AKs. Their results showed that both ALA-PDT and MAL-PDT caused a significant reduction in the number of AK foci, with no significant difference in efficacy [20]. Fu et al., in a recent meta-analysis investigating the combination of PDT with BF-200 ALA (a 5-aminolevulinic acid nanoemulsion of ALA) versus MAL, indicated that PDT with the former (i.e., BF-200 ALA) had a 9% better chance of complete clearance of AK lesions at 3 months of treatment and a 24% better chance of grade II-III AK lesion clearance in comparison to the results of PDT with the use of the latter (i.e., MAL) [21]. In our study, the clearance assessed 12 weeks after treatment completion demonstrated similarly high PDT efficacy (clearance above 80%), regardless of the applied Ps (*p* > 0.05).

PDT is generally considered to give good cosmetic results with high patient satisfaction. This seems to be of particular importance in the treatment of the lesions localized on the exposed parts of the body [22]. In our study, 94% of the patients found the overall cosmetic effect to be excellent, while the remaining 6% assessed it as good, regardless of the used formulation. A study by Ko et al. compared PDT performed with ALA-HCl as well as MAL in AK patients. The cosmetic outcome after the treatment with the former was rated by 90% of the patients as excellent, while PDT performed with the latter was assessed as excellent by 97% of the study participants, which is in agreement with our results. The excellent result was still observed 12 months after the completion of treatment [23]. Räsänen et al. [24] compared the cosmetic outcome of PDT with the use of either BF-200 ALA or MAL in 69 patients with numerous AK lesions. In their study, 12 months after treatment completion, the cosmetic outcome was excellent or good in >90% of the studied patients, regardless of the used PS.

In order to make sure that PDT is highly effective, the following components need to be present, i.e., proper light energy source, oxygen and a PS with desirable properties such as water solubility, lipophilicity, penetration capability and accumulation in the skin.

In order to demonstrate the accumulation of the investigated PSs in the treated skin area, photodynamic diagnostics with white and violet LED diodes emitting LED light may be used [25]. This type of diagnostic involves the use of PpIX fluorescence, thereby enabling not only determination of PS accumulation in the diseased tissue but also establishing the boundaries between healthy and diseased tissues [26] and the extent and nature of the neoplastic skin lesions, malignant and non-malignant [27].

The intensity of red fluorescence depends on the skin penetration by a selected PS and production of PpIX in the epidermis determined by its lipophilic character. The calculated LogP values for the tested photosensitizers indicate a higher lipophilicity of MAL-HCl (ACD/LogP = −0.57, ALogPs = −1.30, miLogP = −1.62) as the ALA methyl ester compared to ALA-HCl and ALA-P (for both substances: ACD/LogP = −0.93, ALogPs = −2.85, miLogP = −1.93). In our study, confluent, intensive fluorescence seen in the ALA-HCl and MAL-HCl-treated skin areas indicates a remarkably fine accumulation of ALA in the skin and a high amount of PpIX production. However, the lower and blotchy fluorescence we observed in the skin areas treated with ALA-P may be suggestive of a more selective accumulation of ALA-P in the AK lesions. Therefore, because of their lower kinetics of PpIX formation, ALA-P may facilitate obtaining a better contrast between the healthy and diseased tissue [28,29]. It seems that the observed differences in the distribution of ALA-P in relation to ALA-HCl and MAL-HCl may result from the presence of the H_2_PO_4_- ion. Available studies suggest that the H_2_PO_4_- ion mediates the proton transfer required for the enolization of amino acids by acting simultaneously as both a general base and a general acid. The dihydro-phosphate ion may catalyze various reactions in proteins, including the racemization of amino acid residues. Therefore, it may contribute to better cellular membrane permeability [30], as racemization of the amino acids influences the functions of many intracellular, extracellular and membrane-bound proteins, and it is considered as a critical factor of protein conformation [31]. However, the observations made must be confirmed in further studies.

PDT is not free of local side effects such as pain, erythema, edema, desquamation, crusting, and pustules, which may occur both during the PDT procedure and in the next hours/days. Urticaria, contact dermatitis, erosive pustular dermatosis of the scalp, pigmentary lesions, scarring, and bullous pemphigoid are less frequently reported [32].

One major drawback of PDT is pain, which may be the reason for discontinuation of the treatment altogether and which may discourage the patient from undergoing future treatments [26]. A number of studies have compared the intensity of pain experienced after application of ALA-HCl or MAL. The results, however, are difficult to interpret due to the use of different formulations and study protocols [33]. Therefore, bearing in mind the intensive pain accompanying PDT, the PDT procedure should be divided into a few stages with time intervals. In our study, which consisted of 22 patients, 16 of them qualified for the second PDT procedure because of the extensiveness of their AK lesions. Although all 22 patients completed the first PDT procedure successfully, one of the 16 patients requiring the second PDT procedure refused further treatment because of unbearable pain. We observed that the intensity of pain on a 10-point VAS scale during and shortly after the first PDT procedure performed in the skin areas treated with either ALA-HCl or MAL-HCl was similar (7.0 on average), whereas it was slightly lower in the skin areas treated with ALA-P (5.8 on average). However, 7 days after the first PDT procedure, the intensity of pain was significantly lower in the areas treated with MAL-HCl or ALA-P (1.4 and 1.3 on average, respectively) than in the areas treated with ALA-HCl (1.8 on average). In most of the studies, patients reported more acute pain at the site of ALA-HCl application [20,34,35,36,37,38,39]. However, in the reports by Yazdanyar et al., who compared the pain response to ALA-HCl and MAL treatment of the scalp and forehead AK lesions, no significant differences in pain intensity between these two formulations were found either during or 30 min after the completion of treatment [40]. Ibotson et al. [41] indicated that it was still unclear which of the tested compounds, i.e., ALA-HCl or MAL, caused more severe pain during irradiation.

In our study, the intensity of pain during the second PDT procedure and 7 days after its completion did not significantly differ regardless of the used PS, and it was slightly lower than during the first PDT procedure (approximately 5 points on average and approximately 1 point on average, respectively). It appears that the pain experienced during the second PDT procedure is better tolerated because of its foreseeable nature.

A limitation of our study is the quality of images showing intensity and distribution of fluorescence in the skin areas after application of ALA-HCl, MAL-HCl, ALA-P. Future studies, e.g., using an animal model and a higher-resolution device are needed.

## 5. Conclusions

ALA-P, a new PS first tested in our study turned out to be similarly effective as ALA-HCl and MAL-HCl. We suggest that the use of ALA-P in PDT of AK lesions should be further investigated, because despite in a small number of our study subjects, the obtained results are promising enough to acknowledge the fact that this new ALA photosensitizer may be an advantageous alternative to the already used ALA-HCl and MAL since its application appears to be slightly less painful and better tolerated.

## Figures and Tables

**Table 1:** Cosmetic outcome of photodynamic therapy assessment.

Grade	Definition
Poor	extensive occurrence of scarring, atrophy, or induration
Fair	slight to moderate occurrence of scarring, atrophy, or induration
Good	no scarring, atrophy, or induration, moderate redness or increase in pigmentation compared with adjacent skin
Excellent	no scarring, atrophy, or induration, slight or no redness or change in pigmentation compared with adjacent skin

**Table 2:** Characteristics of the studied actinic keratosis patients.

Variable	Category	Parameter	Estimate
Age	years	Min-max	60–84
Sex	male	n (%)	21 (95%)
	female		1 (5%)
Localizations of lesions	face	n (%)	4 (18%)
	scalp		18 (82%)
Thickness grade, according to Olsen et al. [14]	I grade (thin)	n (%)	9 (24%)
	II grade (moderately thick)		18 (49%)
	III grade (thick)		10 (27%)

## Data Availability

Data sharing not applicable.

## Supplementary Materials

The following are available online at https://www.mdpi.com/article/10.3390/pharmaceutics14020346/s1, Figure S1: Comparisons of the skin areas in the studied patients before PDT and 12 weeks after procedure completion.
