# Supplementary material for: Topical Photodynamic Therapy with Different Forms of 5-Aminolevulinic Acid in the Treatment of Actinic Keratosis
Source: Pharmaceutics. 2022 Feb 1;14(2):346. doi: 10.3390/pharmaceutics14020346 (PMC8876011; doi:10.3390/pharmaceutics14020346)
Supplement: Supplementary file 1 [file pharmaceutics-14-00346-s001.zip › pharmaceutics-1541213-supplementary.pdf]

pharmaceutics

MDPI

Supplementary Materials: Topical Photodynamic Therapy with Different Forms of 5-Aminolevulinic Acid in the Treatment of Actinic Keratosis

Joanna Bartosińska, Paulina Szczepanik-Kułak, Dorota Raczkiewicz, Marta Niewiedzioł, Agnieszka Gerkowicz, Dorota Kowalczuk, Mirosław Kwaśny and Dorota Krasowska

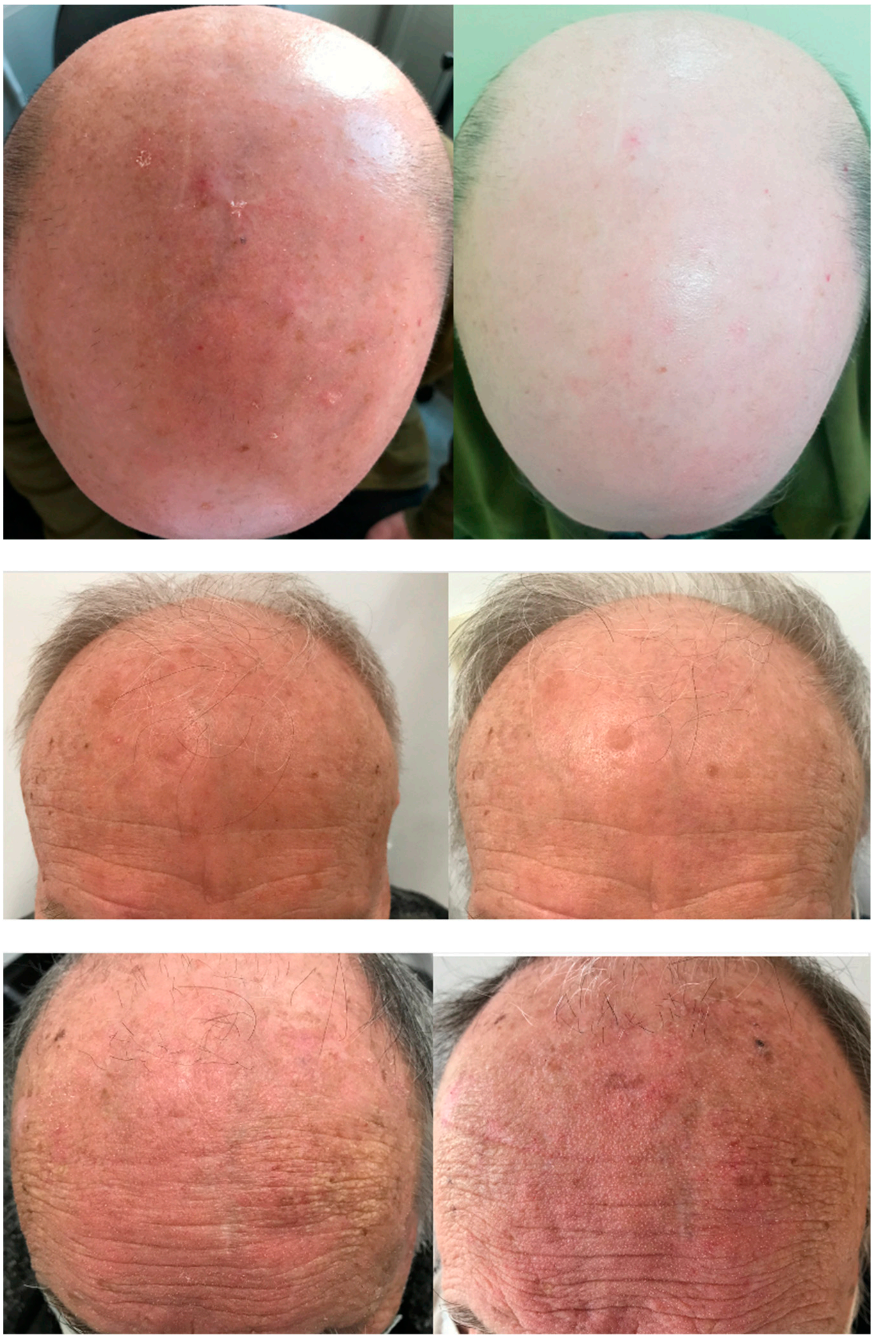

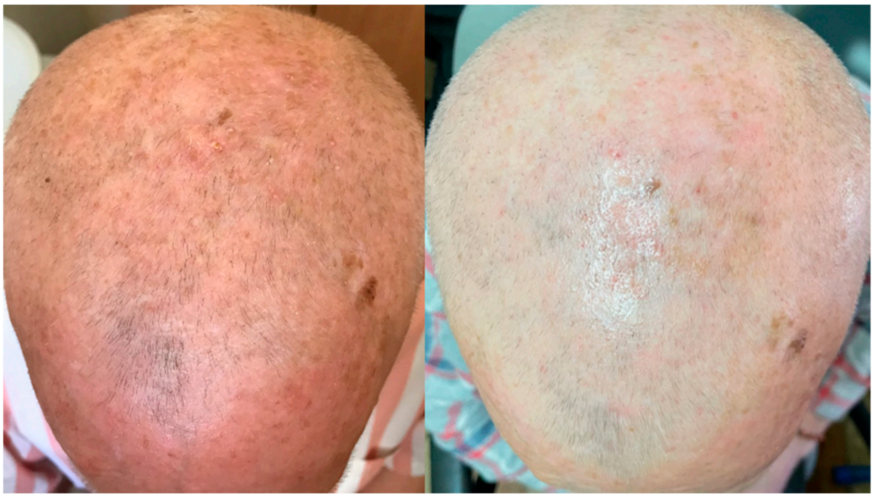

Figure S1. The comparisons of the skin areas in the studied patients before PDT and 12 weeks after the procedure completion.
